# Utilization of somatic healthcare in Croatian patients with schizophrenia spectrum disorder, major depression, PTSD and the general population

**DOI:** 10.1186/s12888-019-2190-8

**Published:** 2019-06-28

**Authors:** Vjekoslav Jeleč, Žarko Bajić, Ivona Šimunović Filipčić, Ivana Portolan Pajić, Mirela Šentija Knežević, Ivan Miloloža, Delfa Radić-Krišto, Tomislav Benjak, Nenad Jakšić, Marina Šagud, Wei Wang, Igor Filipčić

**Affiliations:** 1 Department of Neurosurgery, Dubrava University Hospital, Zagreb, Croatia; 2 City Office for Health, Zagreb, Croatia; 3 Psychiatric Hospital “Sveti Ivan”, Zagreb, Croatia; 4 Department of Psychological Medicine, University Hospital Center Zagreb, Zagreb, Croatia; 5 Faculty of Dental Medicine and Health, Josip Juraj Strossmayer University of Osijek, Osijek, Croatia; 6 Division of Hematology, Merkur University Hospital, Zagreb, Croatia; 7 Faculty of Medicine, Josip Juraj Strossmayer University of Osijek, Osijek, Croatia; 8 Croatian Institute of Public Health, Zagreb, Croatia; 9 Department of Psychiatry, University Hospital Center Zagreb, Zagreb, Croatia; 10 School of Medicine, University of Zagreb, Zagreb, Croatia; 11 Department of Clinical Psychology and Psychiatry, Zhejiang University College of Medicine, Hangzhou, China

**Keywords:** Schizophrenia spectrum disorder, Major depressive disorder, Posttraumatic stress disorder, General population, Specialist health care utilization

## Abstract

**Background:**

Utilization of somatic healthcare services is highly predictive of the development of chronic physical illnesses and increased mortality risks. The objective of this study was to assess the differences in healthcare utilization among patients with schizophrenia spectrum disorders (SSD), major depressive disorder (MDD) and posttraumatic stress disorder (PTSD) and the general population in Croatia.

**Methods:**

We enrolled 566 Croatian participants from the general population, 282 with SSD, 178 with MDD, and 86 with PTSD. The primary outcome was a self-reported specialist consultation for non-psychiatric (e.g., somatic) causes within the previous 12 months.

**Results:**

Although SSD patients with chronic physical illnesses were significantly more often hospitalized for physical illness than the general population, the proportion of patients who had a specialist consultation were equal in SSD and the general population. MDD and PTSD patients had significantly higher adjusted odds for specialist consultation than the general population and SSD patients (MDD compared to SSD: OR = 2.14; 95% CI 1.27–3.59; PTSD compared to SSD: OR = 2.03; 95% CI 1.00–4.10).

**Conclusions:**

SSD patients’ utilization of somatic healthcare is equal to the general population, despite their increased healthcare needs. However, their utilization is lower than in MDD and PTSD patients and, therefore, probably not adequate.

**Trial registration:**

The study protocol was registered at ClinicalTrials.gov (NCT02773108) on May 16, 2016.

## Introduction

It has been known for over two decades that the lifespan of persons with schizophrenia spectrum disorder (SSD) is reduced for 15–20 years, [1] primarily due to a high prevalence of common preventable and/or treatable chronic physical illnesses (CPI) and poor primary care utilization. [2–6] However, even in three Scandinavian countries that provide among the best-quality and most equitably distributed healthcare in the world, this mortality gap has narrowed only by a modest extent and remains stubbornly wide. [7, 8] Croatia has a universal healthcare system, its population is covered by a basic health insurance plan provided by statute and optional insurance administered by the Croatian Health Insurance Fund (CHIF). This means that every citizen has the same accessibility to healthcare. [9] The comorbidity of SSD and CPI has been demonstrated in studies indicating that individuals with SSD are at significantly increased risk of developing CPI, at an earlier age due to both maladaptive health risk behaviors, such as smoking and sedentary lifestyle, and the physiological effects of their psychiatric illnesses. [10–12] Furthermore, the CPI may affect treatment outcomes in SSD and are associated with increased costs. [13–16] It is thus essential not only to manage the symptoms of schizophrenia but also to treat comorbid physical illnesses [17] as physical healthcare needs of SSD patients are elevated. Some studies indicated that the access and utilization of different non-psychiatric health care services in this population is below the general population (GEP). [18–20] However, other studies concluded that no relevant differences in the frequency of SSD patients’ contacts with healthcare professionals exist [21], but that the patterns of health care utilization are different. It was hypothesized that SSD patients have higher rates of hospitalizations although no more frequent contacts with primary and secondary care clinicians. The higher rates of hospitalizations were explained by the delayed first presentation to primary care resulting in patients’ medical conditions being at a more advanced stage, necessitating hospitalization. [6] Finally, at least one study found that a higher number of CPI comorbid to SSD may even be associated with a better quality of health care. [22] The most common approach in comparing health care utilization and the quality of care in SSD patients is the direct comparison of health care utilization or quality measures. For example, the annual frequency of specialist consultations between SSD and GEP is directly compared. However, due to the higher health care needs of SSD population, this approach may lead to erroneous conclusions. Several studies have found the apparent paradox of equality of health services between SSD patients and GEP, but still premature SSD patients’ mortality. [23, 24] Therefore, the equality of health care utilization between SSD and GEP should be interpreted as an inadequate assessment of SSD patients’ medical healthcare needs. The methodological difficulty with such an approach is in defining the optimal health care utilization levels in SSD. We used a surrogate solution and included two additional control groups besides GEP: a group of patients diagnosed with major depressive disorder (MDD) and a group diagnosed with posttraumatic stress disorder (PTSD). Our rationale was that SSD patients’ physical healthcare needs are not lower than in these two psychiatric patient groups. We hypothesized that the targeted “optimal” health care utilization level should not only be higher than in GEP but equal to or higher than in MDD and PTSD. The objective of our analysis was to assess the differences in health care utilization between SSD patients and GEP, compared to patients diagnosed with MDD and PTSD.

## Methods

### Study design

We performed this analysis on the samples from two cross-sectional studies. The first one was the prospective cohort study named “Somatic Comorbidities in Psychiatric Patients (SCPP),” and was performed at Psychiatric Hospital “Sveti Ivan”, Zagreb, Croatia in 2016. The study protocol was registered at ClinicalTrials.gov (NCT02773108) and was approved by the Ethics Committee of Psychiatric Hospital “Sveti Ivan.” All patients signed an informed consent form. The study complied with the World Medical Association Declaration of Helsinki 2013. [25] The second study was a European health interview survey (EHIS) conducted for the first time in the Republic of Croatia between 2014 and 2015, as in all EU Member States, Iceland and Norway according to the European Commission Regulation 141/2013 [26, 27].

### Study population

The study populations were patients diagnosed with SSD (ICD-10, F20-F29), MDD (ICD-10, F32–33, and PTSD (ICD-10, F43.1) (Using the clinical ICD-10 psychiatric diagnosis coded as the principal diagnosis), treated in the psychiatric hospital, and the GEP with the permanent residency in the city of Zagreb and Zagreb County, living in private households. Psychiatric Hospital “Sveti Ivan” covers the same geographic region, although a smaller number of patients came from other parts of the country. The inclusion criteria common to all four targeted populations were: age 18–65. The common exclusion criteria were the inability to answer the questionnaires by themselves. The exclusion criteria for the sample from the psychiatric population were dementia, mental retardation, acute psychosis, and intoxication. 

### Sample types

Samples from SSD, MDD and PTSD populations were one-stage consecutive samples. We consecutively included all patients, that is the entire available population of patients who satisfied the inclusion and exclusion criteria and who were present at the hospital during the enrollment period, either because of being hospitalized or because they came for an outpatient examination. For the general population sample, we used a two-stage, stratified random sample from the EHIS study. The sample frame was based on the Croatian Census 2011 and was designed by the Croatian Bureau of Statistics [26, 27]. The primary sampling unit was the household. Within each household, all present household members were interviewed. Because the members of the same household are not independent concerning our outcomes, the design effect should be assessed. As large number of primary sampling units was selected (more than 600 households), the design effect on the effective sample size was negligible. The overall response rate in EHIS study was 83%. The sociodemographic structure of non-responders was not available to us. The response rate in the study on psychiatric patients was 94%.

### Outcomes

The primary outcome was the self-reported specialist consultation for non-psychiatric causes, experienced within 12 months prior to enrollment. The question was phrased in the same way, and according to the EHIS questionnaire: “When was the last time you consulted a medical specialist because of your needs?” Secondary outcomes were self-reported measurements of blood pressure, cholesterol, blood glucose, pap cervical screening and mammography during the 12 months before the enrollment, use of non-psychiatric medicines within 2 weeks before the enrollment, and the hospitalization because of physical illness during the previous 12 months. These questions were phrased according to the EHIS questionnaire: “When was the last time that a healthcare professional measured your blood pressure…blood cholesterol…etc”.

### Independent variable

The independent variable was a psychiatric diagnosis (SSD, MDD, PTSD), and the GEP. When the patient had both SSD and MDD or PTSD and MDD comorbidities, we grouped her/him according to the main diagnosis and counted the other one only in the number of psychiatric comorbidities. We defined the “main diagnosis” as the older one, the one that was diagnosed first.

### Possible confounders

Pre-planned confounders whose effects we controlled by the multivariable analysis were gender, age, marital status, working status, body mass index, self-perceived general health, self-reported hospitalization for physical illness during the 12 months before the enrollment, and number of CPI. We operationalized the CPI according to EHIS questionnaire where the participants were asked about 15 CPI: 1) asthma (allergic asthma included), 2) chronic bronchitis, chronic obstructive pulmonary disease, emphysema, 3) myocardial infarction (heart attack) or chronic consequences of myocardial infarction, 4) coronary heart disease or angina pectoris, 5) high blood pressure (hypertension), 6) cerebrovascular insult (cerebral hemorrhage, cerebral thrombosis) or chronic consequences of stroke, 7) arthrosis (arthritis excluded), 8) low back disorder or other chronic back defects, 9) neck disorder or other chronic neck defects, 10) diabetes mellitus, 11) allergy, such as rhinitis, hay fever, eye inflammation, dermatitis, food allergy or other allergies (allergic asthma excluded), 12) cirrhosis of the liver, 13) urinary incontinence, problems in controlling the bladder, 14) kidney disease, 15) obesity defined as body mass index (kg/m^2^) ≥30.0. [26] Data were collected by self-completion of the 2nd wave European Health Interview Survey (EHIS). [26] The chronicity of the targeted illnesses was emphasized by the introduction: “Here is the list of chronic illnesses or conditions”, by show-cards with the instruction for the respondent written on it: “Mark with “yes” or “no” for every chronic illness”, and by the names of CPI that contain the word: “chronic” when there may be some ambiguity: “chronic bronchitis”, “chronic consequences of myocardial infarction”, “chronic consequences of stroke”, “low back disorder or other chronic back defects”, “neck disorder or other chronic neck defects”.

### Statistical analysis

The primary analysis was performed using a multivariable binary logistic regression in the per-protocol population of participants with complete data on the primary outcome and all pre-planned possible confounders. We analyzed the differences in available sociodemographic and clinical characteristics of patients with and without missing data on the primary outcome and all preplanned confounders. As the standardized effect size measures, we presented odds ratios with their 95% confidence intervals (CI). Level of statistical significance was set at a two-tailed *p* < 0.05, and all CI was given at the 95% level. The analysis was carried out using the NCSS 12 Statistical Software (2018) (NCSS, LLC. Kaysville, Utah, USA).

## Results

### Participants

**Fig. 1:**
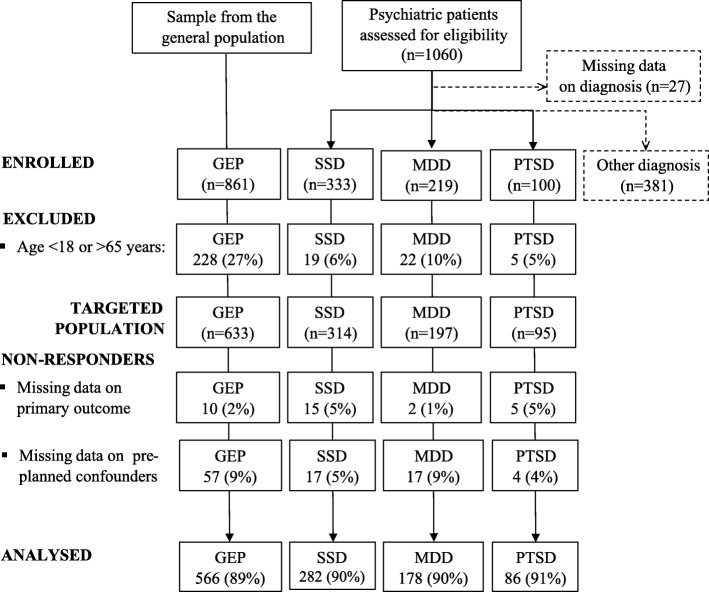
Participants flow

We assessed 1060 psychiatric patients for eligibility (Fig. 1). From this sample and the sample of 861 from the general population, we excluded participants younger than 18 and older than 65. There were 127 patients with no data on the primary outcome and pre-planned possible confounding factors, 67 of them from the general population and 60 from the population of psychiatric patients. Percentages of non-responders were similar in the samples from all four targeted populations: 11% in GEP, 10% in SSD and MDD, and 9% in PTSD. Non-responders were comparable to responders according to gender, marital status, and prevalence of obesity. Median (IQR) age of non-responders and responders were 40 (33–51) vs 45 (31–55) in GEP, 42 (34–55) vs 40 (32–50) in SSD, 54 (48–57) vs 52 (45–57) in MDD, and 56 (50–62) vs 53 (46–57) in PTSD patients. In GEP non-responders vs responders there were CPI in 43% vs 57%, in SSD 63% vs 65%, in MDD 74% vs 84%, and in PTSD 67% vs 91% of cases. Finally, we analyzed 546 psychiatric patients diagnosed with SSD, MDD and PTSD, and 566 patients from the GEP. In the sample from the population of patients diagnosed with SSD 113 (40%) was diagnosed with schizophrenia (ICD-10 F20), 70 (25%) with acute and transient psychotic disorder (ICD-10 F23), 46 (16%) with unspecified nonorganic psychosis (ICD-10 F29), 44 (16%) with schizoaffective disorder (ICD-10 F25) and 9 (3%) with other psychosis. Patients with PTSD were predominantly war veterans, 72 (84%). There were some relevant differences between samples from our four targeted populations whose possible confounding effects we controlled for using a multivariable analysis (Table 1).

**Table 1:** Participants’ sociodemographic and clinical characteristics

	GEP (*n* = 566)		SSD (*n* = 282)		MDD (*n* = 178)		PTSD (*n* = 86)	
Gender								
men	263	(46.5)	159	(56.4)	74	(41.6)	72	(83.7)
women	303	(53.5)	123	(43.6)	104	(58.4)	14	(16.3)
Age (years), median (IQR)	45	(31–55)	40	(32–50)	52	(45–57)	53	(46–57)
Education								
primary	64	(11.3)	36	(12.8)	20	(11.2)	9	(10.5)
secondary	345	(61.0)	175	(62.1)	114	(64.0)	61	(70.9)
university	157	(27.7)	71	(25.2)	44	(24.7)	16	(18.6)
Marital status								
single	165	(29.2)	191	(67.7)	38	(21.3)	17	(19.8)
married	354	(62.5)	58	(20.6)	93	(52.2)	43	(50.0)
widowed or divorced	47	(8.3)	33	(11.7)	47	(26.4)	26	(30.2)
Working status								
employed	389	(68.7)	124	(44.0)	103	(57.9)	28	(32.6)
unemployed	77	(13.6)	81	(28.7)	27	(15.2)	12	(14.0)
retired	100	(17.7)	77	(27.3)	48	(27.0)	46	(53.5)
Body mass index (kg/m^2^), median (IQR)	25	(22–28)	26	(24–30)	27	(24–31)	28	(25–31)
Regular smoking of tobacco	208	(36.7)	161	(57.1)	96	(53.9)	44	(51.2)
Self-perceived general health (bad)	52	(9.2)	44	(15.6)	63	(35.4)	50	(58.1)
Number of physical comorbidities, median (IQR)^a^	1	(0–2)	1	(0–2)	2	(1–4)	3	(1–4)
Chronic physical comorbidities								
none	268	(47.3)	120	(42.6)	39	(21.9)	12	(14.0)
one	121	(21.4)	70	(24.8)	33	(18.5)	11	(12.8)
two or more (multimorbidity)	177	(31.3)	92	(32.6)	106	(59.6)	63	(73.3)
Being hospitalized for physical illness during the last 12 months	44	(7.8)	78	(27.7)	54	(30.3)	24	(27.9)
Days of hospitalization for physical illnesses annually, median (IQR)^b^	4	(2–10)	30	(9–94)	20	(8–90)	10	(2–99)
Status at enrollment								
outpatients			125	(44.8)	96	(55.5)	35	(41.7)
in-patients			136	(48.7)	52	(30.1)	39	(46.4)
daily hospital			18	(6.5)	25	(14.5)	10	(11.9)
Duration of primary illness (years), median (IQR)			7	(1–15)	4	(1–8)	10	(4–15)
Psychiatric comorbidity			89	(31.6)	139	(78.1)	73	(84.9)
Previous psychiatric hospitalizations			3	(1–7)	1	(0–3)	3	(1–7)
Psychiatric treatment at enrolment								
antipsychotics			262	(92.9)	69	(38.8)	43	(50.0)
antidepressants			109	(38.7)	162	(91.0)	72	(83.7)
benzodiazepines			210	(74.5)	162	(91.0)	83	(96.5)

Data are presented as number (percentage) of participants if not stated otherwise

*Abbreviations*: *GEP* general population, *SSD* schizophrenia spectrum disorder, *MDD* major depressive disorder, *PTSD* posttraumatic stress disorder, *IQR* interquartile range

^a^Excluding obesity (BMI ≥ 30)

^b^Only participants who were hospitalized at least once

### Specialist consultations

**Fig. 2:**
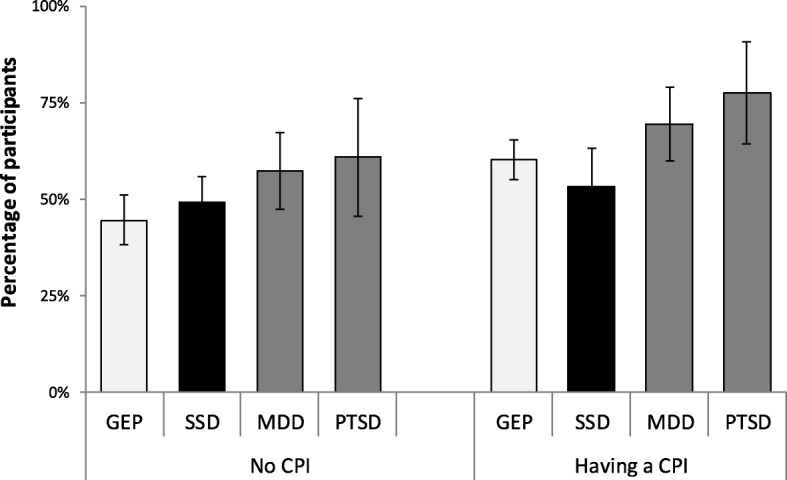
Adjusted percentage of participants having a specialist consultation for non-psychiatric illness during the 12 months before the enrollment; error lines represent 95% confidence intervals; adjustment was done for: gender, age, marital status, working status, body mass index, self-perceived general health, number of physical illnesses, hospitalization during the 12 months before the enrollment

The proportion of patients who had a specialist consultation for non-psychiatric reasons was equal in SSD and GEP (Table 2). After the adjustment for all pre-planned possible confounding factors, the odds ratio for the consultation was not significantly different between these two samples. MDD and PTSD patients had significantly higher adjusted odds for specialist consultation than GEP (Table 2) and significantly higher adjusted odds than SSD patients (MDD: OR = 2.14; 95% CI 1.27–3.59; PTSD: OR = 2.03; 95% CI 1.00–4.10). In patients with CPI, MDD and PTSD patients had significantly higher adjusted odds for specialist consultation than GEP with CPI (MDD: OR = 1.91; 95% CI1.02–3.57 and PTSD: OR = 2.62; 95% CI 1.06–6.45) (Fig. 2). Both MDD and PTSD patients had significantly higher adjusted odds for specialist consultation than SSD patients (MDD: OR = 5.61; 95% CI 2.22–14.21; PTSD: OR = 6.11; 95% CI 1.81–20.58). There were no significant differences in the odds for specialist consultation between GEP, SSD, MDD, and PTSD with no CPI (Fig. 2).

**Table 2:** Consultation with specialists for non-psychiatric illness during the previous 12 months

	Had a consultation		95% (CI)	Unadjusted		Adjusted^a^, multivariable		
				OR	(95% CI)	OR	(95% CI)	p
GEP	283	(50.0)	(46–54)	1		1		
SSD	141	(50.0)	(44–56))	1.00	(0.75–1.33)	0.89	(0.61–1.28)	0.520
MDD	132	(74.2)	(67–80)	2.87	(1.98–4.17)	1.64	(1.04–2.59)	0.033
PTSD	64	(74.4)	(64–83)	2.91	(1.74–4.85)	2.29	(1.20–4.37)	0.012
Gender								
men	256	(45.1)	(41–49))	1		1		
women	364	(66.9)	(63–71)	2.47	(1.93–3.14)	3.01	(2.26–4.02)	< 0.001
Age				1.02	(1.01–1.03)	0.99	(0.98–1.01)	0.316
Education								
primary	72	(55.8)	(47–65)	1		1		
secondary	384	(55.3)	(52–59)	0.98	(0.67–1.43)	1.13	(0.72–1.78)	0.583
university	164	(56.9)	(51–63)	1.05	(0.69–1.59)	1.44	(0.88–2.37)	0.151
Marital status								
single	188	(45.7)	(41–51)	1		1		
married	339	(60.2)	(56–64)	1.80	(1.39–2.33)	1.24	(0.84–1.83)	0.284
widowed or divorced	102	(66.7)	(59–74)	2.37	(1.61–3.50)	1.20	(0.72–2.01)	0.487
Working status								
employed	348	(54.0)	(50–58)	1		1		
unemployed	101	(51.3)	(44–58)	0.90	(0.65–1.23)	0.84	(0.57–1.24)	0.390
retired	171	(63.1)	(57–69)	1.45	(1.09–1.95)	0.84	(0.57–1.26)	0.842
Body mass index (kg/m^2^)				1.04	(1.01–1.06)	1.02	(0.99–1.05)	0.249
Self-perceived general health								
good or average	452	(50.1)	(47–53)	1		1		
bad	168	(80.4)	(74–86)	4.09	(2.84–5.89)	2.14	(1.38–3.31)	0.001
Having a CPI	377	(68.3)	(64–72)	2.95	(2.30–3.78)	1.76	(1.27–2.45)	0.001
Number of physical comorbidities				1.51	(1.39–1.65)	2.14	(1.38–1.37)	< 0.001
Being hospitalized for physical illness during the last 12 months	162	(81.0)	(75–86)	4.23	(2.90–6.16)	3.68	(2.40–5.64)	< 0.001

Data are presented number (percentage; percentage’s 95% CI) of participants

*Abbreviations*: *OR* odds ratio, *CI* confidence interval, *p* two-tails statistical significance calculated using multivariable binary logistic regression, *GEP* general population, *SSD* schizophrenia spectrum disorder, *MDD* major depressive disorder, *PTSD* posttraumatic stress disorder, *IQR* interquartile range, *CPI* chronic physical illness

^a^Adjustment was done for: gender, age, marital status, working status, body mass index, self-perceived general health, number of physical illnesses, hospitalization during the 12 months before the enrollment

### Diagnostic and control testing

Compared to 217 (69%) of GEP with CPI who measured blood pressure at least once during the 12 months before the enrollment, SSD and MDD patients had higher adjusted odds for blood pressure measurement (SSD: OR = 3.61; 95% CI 1.55–8.40; MDD: OR = 5.38; 95% CI 2.01–14.44). Blood pressure was measured in 73 (87%) of SSD patients, and 88 (95%) of MDD patients. Measurement of cholesterol, blood glucose, gynecological pap test or mammography was not significantly different in any group with CPI. In patients with no CPI, blood pressure was measured at least once in 162 (83%) SSD patients, and they had more than 3.5 times higher adjusted odds for blood pressure measurement than GEP with no CPI (OR = 3.61; 95% CI 1.55–8.40). Compared to GEP with no CPI, SSD and MDD patients had higher adjusted odds for blood glucose testing (SSD: OR = 3.02; 95% CI 1.79–5.08; MDD: OR = 2.21; 95% CI 1.10–4.44). Blood glucose tests were performed in 131 (68%) SSD patients with no CPI. The difference between PTSD and GEP was not significant (OR = 1.20; 95% CI 0.47–3.11). Similar results were obtained for cholesterol measurements. Both MDD and SSD patients had higher adjusted odds for lipid level assessment compared to GEP (MDD: OR = 2.30; 95% CI 1.38–3.83; SSD: OR = 2.75; 1.38–5.46), which was performed in 115 (60%) of SSD patients with no CPI. Again, PTSD patients with no CPI had no significantly different adjusted odds for cholesterol testing compared to GEP with no CPI. The interaction of any study group and current, regular smoking on testing for blood pressure, cholesterol or blood glucose testing was not significant. Pap cervical screening and mammography were not performed significantly more or less frequently in psychiatric women patients’ samples with no CPI than in GEP. Pap test was done in 39 (56%) of women SSD patients with no CPI. Mammography was performed in 24 (34%) of women SSD patients with no CPI.

### Medicines usage

The difference in non-psychiatric medicine usage during 2 weeks before the enrollment was not significantly different between GEP with CPI: 208 (65%) and SSD: 51 (61%), MDD: 68 (73%) or PTSD patients with CPI: 39 (78%). On this particular sample level, all three psychiatric patient groups had non-significantly lower odds for non-psychiatric medicine usage compared to GEP (SSD: OR = 0.0.66; 95% CI 0.35–1.23; MDD: OR = 0.83; 95% CI 0.46–1.50; PTSD: OR = 0.74; 95% CI 0.32–1.73).

### Hospitalizations for physical illnesses

SSD patients with CPI were significantly more likely to be hospitalized for physical illness than GEP. Adjusted odds for at least one hospitalization was almost four times higher in SSD than in GEP group (OR = 3.96; 95% CI 2.02–7.76). SSD patients with CPI were hospitalized in 33 (39%) of cases, and GEP in 40 (13%). The other two psychiatric groups, MDD and PTSD, were hospitalized in 29 (30%), and 18 (35%) respectively. After the adjustment for age, gender, education, working and marital status and body mass index SSD patients with CPI had significantly higher odds for hospitalization for physical illness then MDD patients (OR = 0.63; 95% CI 0.42–0.94), but not compared to PTSD patients (OR = 0.91; 95% CI 0.73–1.14).

## Discussion

Although SSD patients with or without CPI have higher specialist healthcare needs than GEP, our study found that their specialist physical healthcare utilization is not higher. This finding is not psychiatric-specific, but SSD specific, because patients diagnosed with MDD or PTSD use specialist health care to larger extent than GEP or SSD patients. SSD patients’ utilization of somatic healthcare is equal to GEP, despite their increased healthcare needs. Moreover, this utilization is lower than in MDD and PTSD patients and, therefore, likely to be inadequate.

Our finding that the hospitalization rate and its median duration are increased in SSD patients, whereas their consultation with the specialist is equal or lower than in GEP, is consistent with the conclusion of previous studies that SSD patients have different patterns of health care utilization, not seeking help until their condition requires hospitalization. [21, 24] This delay in contact with the health care system may be caused by SSD patients lower ability to interpret the physical symptoms correctly, lower pain sensitivity caused by antipsychotics [3], lower education and social support, socioeconomic status, self-stigmatization or inactivity, and loss of initiative due to negative symptoms. [28] If the health care system requires a patient’s self-initiative to be fully utilized in order to access care [29] then SSD patients would not have equal chances for the health care utilization and CPI treatment when compared to GEP, or to patients diagnosed with MDD or PTSD whose cognitive executive functioning is less affected by the primary psychiatric condition. [30] MDD and PTSD patients also have better socioeconomic status, better social support, and lower self and social stigmatization. Also, MDD, PTSD and particularly GEP patients’ quality of communication with the somatic health care clinicians may be superior to the communication skills of SSD patients [31–33]. On the other hand, the incidence of MDD is increased in many different medical conditions. [34] Depression and anxiety are the most often co-occurring mental disorders with medical illnesses and are independent predictors of worsened outcome in numerous medical conditions including diabetes and cardiovascular disease. [35] As a consequence, different somatic medicine specialists are more accustomed to MDD and more knowledgeable about MDD than about SSD. Our finding that PTSD, predominantly veteran population, have the highest utilization of specialist healthcare is concordant with many other studies showing the higher rates of guideline-concordant care in this population. [36] The reach, funding, and efficacy of non-governmental veteran organizations as well as government care, are superior to non-medical organizations aimed at helping patients with SSD in Croatia. Finally, MDD and PTSD patients, as was shown by our study, perceive their health status significantly less favorably than SSD patients, while this perception is the significant predictor of specialist consultation, and is associated with marked increase in use of healthcare. [37]

One of the probable causes of insufficient health care utilization in SSD population has nothing to do with SSD, but with the single-disease paradigm. [38] The specialists, secondary and tertiary healthcare systems are single-disease focused. Specialists are usually highly trained in specific medical conditions, the health care system is fragmented, and multimorbidity requires contacts with different clinicians, different departments or even hospitals. This approach poses an additional burden on SSD patients who may be less able to cope with such a demanding diversity, which may affect their adherence [39], that may further complicate the relationship with the clinicians, the most prevalent cause of psychiatric treatment failure [40, 41], and physical and mental health care are most often physically separated [29], and as competent and comprehensive psychiatric care often cannot be provided in internal medicine facilities, nor can psychiatric institutions provide effective somatic medicine due to lack of resources. Such a separation of available services may hinder SSD patients more than MDD or PTSD patients.

Contrary to some previous studies’ conclusion that high blood pressure is often missed in several psychiatric populations [42], we found a higher frequency of blood pressure monitoring in SSD and MDD patients than in GEP. This result may be institution-specific as the blood pressure measurement, and complete blood count is routinely performed at each hospital admission in Psychiatric Hospital “Sveti Ivan”, where we enrolled the samples from the psychiatric patient population. Although a significantly higher proportion of SSD patients than participants from the GEP had their blood glucose assessed, the absolute magnitude was too low (68%). If 93% of our participants with SSD were treated with antipsychotics at the time of enrollment and if 100% would be treated with these metabolically active drugs, then the blood glucose and cholesterol measurement should be routinely and regularly performed in all SSD patients regardless of CPI. [43]

### Limitations of the study

The first limitation of our study was the relatively lower reliability of our primary outcome which was based on the self-report questionnaire, and it was not checked independently. As the data were limited to the EHIS study questionnaire, there was no means of excluding this potential source of recall bias. Future studies should use the more precise and comprehensive measures of specialist healthcare utilization taking into the account number of consultations, differences between public and private specialists’ offices, the reasons and outcomes of these consultations, type of medical specialty, extent and differences of specialists’ clinical inertia, that is their occasional deviation from the evidence-based clinical guidelines and the best practices, as well as stigma, care and use of emergencies. Second, we based our assumption on the higher specialist health care needs in SSD than in GEP on participants self-reports and literature however neither may be adequately valid and reliable in this particular case. Future studies should define the specialist’ health care utilization relative to the objectively measured health care needs. Third, we treated SSD patients like one, homogenous group, which is not precisely accurate. Future studies should differentiate specific SSD diagnosis, the severity of illness and configuration of symptoms, and assess the same question more depth. Fourth, we did not record the data on the substance abuse although it may moderate the healthcare utilization and may be different between our targeted populations.

Fifth, one of the exclusion criteria we used to define the targeted populations was the participants ability to answer the questionnaires by themselves. This could exclude patients with more severe CPI symptoms and with the most severe presentations of their psychiatric condition. If this exclusion criterion’s effect on CPI were comparable in our targeted populations, the selection bias would lower the generalizability of both of our samples and the precision of our findings, but not the direction of our main conclusion. If this effect on CPI were stronger in one of our targeted populations, it would jeopardize the internal validity of the study. This effect on the severity of psychiatric condition in principle may have two different consequences: if the excluded patients with the most severe mental disorder have a higher utilization of somatic healthcare, the described limitation would enlarge the difference toward GEP. If the excluded patients utilization of somatic healthcare is lower, this will lower the difference toward GEP. We could not speculate about the exact direction nor the magnitude of this effect.

Sixth, we selected the different sample types from the population of psychiatric patients and the GEP: consecutive and stratified, random respectively. Our consecutive sample might increase the risk of selection bias and result in an overrepresentation of the population with better access to healthcare. If so, this source of bias would act in favor of our null hypothesis and increase the risk of false negative results. We were not able to select the random sample from the total population of psychiatric patients, and only new studies performed on random samples or the consecutive samples but with markedly longer enrollment period may check and probably correct this possible source of lower internal validity of our results. Seventh, we selected the sample from the population of psychiatric patients in the large psychiatric hospital in the highly urban capital of Croatia. Therefore, our results should be cautiously generalized to the population treated in smaller provincial hospitals and psychiatric wards of the general hospitals in less affluential and more rural parts of the country. Eight, despite the fact that we enrolled the patients in hospital wards, daily hospitals, and outpatients offices, and that only a minimal number of patients with SSD, MDD or PTSD in Croatia are diagnosed and having their treatments controlled out of the psychiatric hospital, the population of outpatients was probably somewhat underestimated in our sample. It is likely that the part of the population that is missing in our samples have a more severe disorder, but possibly also better access to healthcare.

## Conclusion

SSD patients’ utilization of somatic healthcare is equal to the general population, despite their increased healthcare needs. Moreover, this utilization is lower than in MDD and PTSD patients and, therefore, likely to be inadequate. These results raise concerns over the adequacy of physical health care for patients diagnosed with SSD. Croatia has universal health coverage that provides more equal opportunities to access care, so in other healthcare systems, the observed inequalities may differ. If one aims to control the increased mortality in SSD population, improve these patients´ quality of life, and effectively reduce the influence of CPI on psychosis treatment outcomes, interventions aimed at facilitating preventive healthcare utilization should be integrated into psychiatric treatment.

## Data Availability

The datasets used and/or analyzed during the current study are available from the corresponding author upon reasonable request.

## Abbreviations

BMI: Body mass index
CHIF: Croatian Health Insurance Fund
CI: Confidence interval
CPI: Chronic physical illness
EHIS: European health interview survey
GEP: General population
ICD: International Classification of diseases
IQR: Interquartile range
MDD: Major depressive disorder
OR: Odds ratio
PTSD: Posttraumatic stress disorder
SCPP: “Somatic Comorbidities in Psychiatric Patients” study
SSD: Schizophrenia spectrum disorder
